# To be or not to be modified: Miscellaneous aspects influencing nucleotide modifications in tRNAs

**DOI:** 10.1002/iub.2041

**Published:** 2019-04-01

**Authors:** Pierre Barraud, Carine Tisné

**Affiliations:** ^1^ Expression génétique microbienne Institut de biologie physico‐chimique (IBPC), UMR 8261, CNRS, Université Paris Diderot Paris France

**Keywords:** tRNA, RNA modifications, modification enzymes, multispecific enzymes, modification circuits, regulation, epitranscriptome, demethylation, demodification, RNA epigenetics, stress response

## Abstract

Transfer RNAs (tRNAs) are essential components of the cellular protein synthesis machineries, but are also implicated in many roles outside translation. To become functional, tRNAs, initially transcribed as longer precursor tRNAs, undergo a tightly controlled biogenesis process comprising the maturation of their extremities, removal of intronic sequences if present, addition of the 3′‐CCA amino‐acid accepting sequence, and aminoacylation. In addition, the most impressive feature of tRNA biogenesis consists in the incorporation of a large number of posttranscriptional chemical modifications along its sequence. The chemical nature of these modifications is highly diverse, with more than hundred different modifications identified in tRNAs to date. All functions of tRNAs in cells are controlled and modulated by modifications, making the understanding of the mechanisms that determine and influence nucleotide modifications in tRNAs an essential point in tRNA biology. This review describes the different aspects that determine whether a certain position in a tRNA molecule is modified or not. We describe how sequence and structural determinants, as well as the presence of prior modifications control modification processes. We also describe how environmental factors and cellular stresses influence the level and/or the nature of certain modifications introduced in tRNAs, and report situations where these dynamic modulations of tRNA modification levels are regulated by active demodification processes. © 2019 IUBMB Life, 71(8):1126–1140, 2019

**Abbreviations**
D: dihydrouridine
H_2_O_2_: hydrogen peroxide
HPLC: high‐performance liquid chromatography
manQ: mannosyl‐queuosine
MMS: methyl methanesulfonate
mRNA: messenger ribonucleic acid
oQ: epoxyqueuosine
pre‐tRNA: precursor of transfer ribonucleic acid
Q: queuosine
RNA: ribonucleic acid
rRNA: ribosomal ribonucleic acid
tRNA: transfer ribonucleic acid
yW: wybutosine
Ψ: pseudouridine

## INTRODUCTION

**Figure 1:**
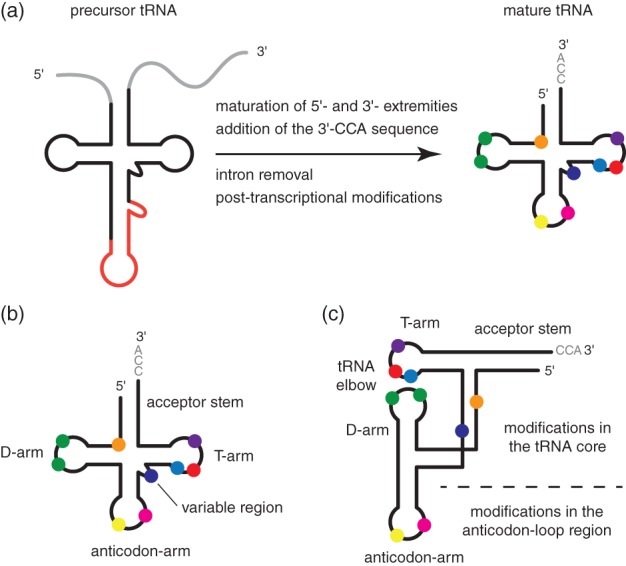
Biogenesis of tRNAs and posttranscriptional modifications. (a) The biogenesis of tRNAs. Precursor tRNAs (*left*) contain 5′‐leader and 3′‐trailer extremities (grey), and some tRNA species also contain introns (red). The different processing steps leading to mature tRNAs (*right*) include the removal of the 5′‐leader and 3′‐trailer sequences, the addition of the 3′‐CCA amino‐acid accepting sequence, the removal of intronic sequences, and the incorporation of a large number of posttranscriptional chemical modifications (filled circles of various colors). (b) Cloverleaf representation of tRNA secondary structure. The five main regions of tRNAs, namely the acceptor stem, the D‐arm, the anticodon‐arm, the variable region, and the T‐arm are labeled on the scheme. Typical modifications are represented with filled circles of various colors. (c) Schematic representation of the L‐shaped three‐dimensional structure of tRNA. In this structure, the acceptor stem stacks on the T‐arm and the D‐arm stacks on the anticodon‐arm. The T‐loop and D‐loop interact and form the tRNA elbow structure. Modifications can be classified into two groups, namely modifications in the tRNA core and modifications found in the anticodon‐loop region.

Transfer RNAs (tRNAs) are essential components of the cellular protein synthesis machineries, but also serve additional functions as broad as amino acids delivery to membrane lipids, synthesis of peptidoglycan, and antibiotics 1, 2. In addition, under stress conditions, some tRNAs are cleaved, creating molecules acting in signaling or as regulators of gene expression 3, 4. To ensure these broad varieties of function within cells, tRNAs undergo a tightly controlled biogenesis process leading to the formation of mature and functional tRNAs. As tRNAs are highly abundant RNA molecules within cells, the tight regulation of their biogenesis is essential to prevent production and accumulation of nonfunctional tRNAs, and thus ineffective cellular energy consumption. The biogenesis of tRNAs starts with the synthesis of a pre‐tRNA produced by the transcription of a tRNA gene. The initial transcript is then subjected to a number of processing steps to yield the mature tRNA molecule (Fig. 1a). Depending on the organism from which the tRNA originates and on the identity of the tRNA, these steps may comprise the removal of the 5′‐leader and 3′‐trailer sequences from the pre‐tRNA transcript, the addition of the 3′‐CCA amino‐acid accepting sequence, the removal of intronic sequences, and the incorporation of a large number of posttranscriptional chemical modifications (Fig. 1a). Overall, these processing events generate a mature tRNA molecule adopting the well‐known cloverleaf secondary structure, defining five main regions called the acceptor stem, the D‐arm, the anticodon‐arm, the variable region, and the T‐arm (Fig. 1b). It is worth noting that these processing events are occurring in an intricate manner and not in a pure stepwise fashion 5, 6, 7. For instance, posttranscriptional modifications can be introduced throughout the entire process, with some modifications being incorporated on pre‐tRNAs with unprocessed 5′‐ or 3′‐extremities, some on pre‐tRNAs with mature extremities but with unprocessed introns, and some on tRNAs with mature extremities and processed introns. The chemical nature of these modifications is highly diverse, with more than hundred different modifications identified in tRNAs 8, 9, 10, 11. Among them, the most frequently found modifications include simple modifications such as ribose or base methylation (e.g., Gm, Cm, m^1^A, m^2^G, m^5^U, m^7^G, m^5^C), base reduction (dihydrouridine, D), base isomerization (pseudouridine, Ψ), and base thiolation (e.g., s^2^U, s^4^U, s^2^C). Other modifications include complex modifications involving the addition of larger chemical groups or requiring multiple modification steps (e.g., t^6^A, m^5^s^2^U, mcm^5^Um, ms^2^i^6^A—for the description of the nomenclature associated with RNA modifications, see for instance 10, 12, 13). The distribution of modifications along the tRNA sequence is far from random, and certain positions are found to be more frequently modified, such as positions 54 and 55 in the T‐arm, 16 and 20 in the D‐arm, and 32, 34, and 37 in the anticodon‐arm, whereas some other positions remain mostly unmodified 14. Although these modifications might seem to be distributed in several regions of the tRNA if one locates them on the cloverleaf secondary structure (Fig. 1b), it is striking to recognize that the three‐dimensional L‐shaped structure of the tRNA, which is formed via intricate interactions between the T‐ and D‐loops, tends to sort the modifications into two groups, namely modifications found in the tRNA core and modifications in the anticodon‐loop region (Fig. 1c). It is worth noting that most modifications found in the tRNA core are simple modifications, whereas most complex modifications are introduced in the anticodon‐loop region of tRNAs. Importantly, all the biological functions of tRNAs in cells are to various extents affected by modifications. For instance, the roles of modifications in the anticodon‐loop region in decoding during protein synthesis are well documented 9. In addition, modifications found in the tRNA core are collectively implicated in the folding and stability of tRNAs. It is now clear that even though most genes encoding tRNA modification enzymes are unessential and strains deleted from these genes show no obvious phenotypes in conditions of optimal growth, modifications in the tRNA core are key for tRNA stability 12, 15. Effects on stability are most of the time revealed by the combination of multiple alterations in the modification genes 16, 17, but may also result from a single lack of modification in a single tRNA, such as the m^1^A58 modification, which is essential for the stability of the yeast initiator tRNA_i_^Met^ 18. Interestingly, the lack of certain specific modifications in the tRNA core has pronounced effect on the stability or function of only a small number of tRNAs, the effect on many others being small to inexistent 19. This suggests that modifications do not have the same beneficial effect for all tRNAs, and that the introduction of certain modifications in some tRNAs is the consequence of the presence of modification enzymes important for other tRNA species 19. In addition to these functions in decoding and stability, modifications in tRNAs are also affecting alternative functions of tRNAs outside translation, making all aspects of tRNA biology controlled and modulated by modifications 1, 4, 9.

Collectively, the introduction of modifications at appropriate positions in tRNAs is of primary importance for optimal cell function. The understanding of the mechanisms that determine and influence nucleotide modifications in tRNAs thus holds a central position in the general understanding of the biological functions of tRNA modifications. Within each organism, although tRNA molecules must all be, to some extent, sufficiently similar to each other to be recognized and used by the translation machinery, they at the same time need to be sufficiently dissimilar to be uniquely recognized by their cognate aminoacyl‐tRNA synthetases. This notion is perfectly appropriate to describe the challenge faced by tRNA modification enzymes, which have been shaped by million years of coevolution with tRNAs to deal with a population of highly similar but unique tRNAs such as to modify them in a very specific manner 20, 21. The present review aims at presenting and summarizing the different aspects that determine whether a certain position in a tRNA molecule is modified or not. These aspects include the sequence and structural determinants of modification enzymes, as well as the presence of prior modifications, and will be presented in the first parts of this review. Importantly, modifications in tRNA are not static decorations that are introduced on every tRNA of the cell in any circumstances. The level of certain modifications in a tRNA population can change dynamically in response to several factors, and in some cases, these dynamic changes are thought to be not only due to alterations in the introduction of these modifications, but also to active demodification processes. These aspects will be described in the latest parts of this review. These elements will be discussed in the light of selected examples from prokaryotic and cytoplasmic tRNAs, and this review will not cover all particularities found in mitochondrial tRNAs. The reader interested in posttranscriptional modifications in mitochondrial tRNAs is thus referred to recent excellent reviews 22, 23.

## Predicting Modifications in tRNA Sequences

The entire modification pattern of a complete set of tRNAs has been experimentally determined only for a small number of organisms, including *Escherichia coli*, *Mycoplasma capricolum*, *Lactococcus lactis*, *Streptomyces griseus*, *Saccharomyces cerevisiae*, and *Holoferax volcanii* 8. Even with the latest technological developments, the experimental determination of the complete modification status of tRNAs remains a difficult and demanding process 24, 25, 26, which can explain in part the relatively low number of organisms, especially in eukaryotes and archaea, for which the complete landscape of tRNA modifications is known. This provided a general interest and a serious motivation for developing computational tools aiming at predicting the nature and the sites of modification in tRNA sequences from any organism. Some of these tools have been developed recently, the most comprehensive computational method for predicting posttranscriptional modifications in tRNAs being tRNAmodpred 27. The predictions performed by tRNAmodpred for organisms for which tRNA modification profiles are unknown but for which the complete sequence of the genome is available, are based on detection of homology to known tRNA modification enzymes conserved during evolution. After the initial identification of tRNA modification genes in the genome, the set of putative enzymes is mapped onto tRNA modification pathways from the MODOMICS database 8, and the resulting potential modifications are then mapped onto the tRNA sequences of interest. The mapping is done based on the identity of the nucleotide present at a given position, meaning that all tRNAs presenting the appropriate nucleotide at the target position of an identified modification enzyme will be marked as modified at this position. In addition, more specific tools aiming at predicting modifications in tRNAs have been developed such as PPUS that is specialized in the identification of pseudouridine sites in yeast and human 28, and tRNAmod that is very efficient in predicting the three most common uridine modifications, namely pseudouridine, dihydrouridine, and 5‐methyluridine 29. Although tRNAmodpred is an attractive computational tool that meets the important need for obtaining information on the nature and the sites of modification in tRNA sequences, it suffers from major limitations, which overall result in a relatively low accuracy of the predictions 27. True experimental determination of modification patterns therefore remains essential. It provides a wealth of information that is valuable per se, and most probably decisive to improve the predictions of computational tools. The most important limitation leading to false positive predictions in tRNAmodpred results from the assumption in the algorithm that all tRNA modification enzymes can modify any tRNA sequence as long as it carries the correct nucleotide at the position to be modified. The method would thus need to be improved by incorporating knowledge on enzyme specificities, as it is well known that tRNA modification enzymes have specificity determinants that control whether a specific tRNA is indeed modified by a given enzyme. The determinants of specificity in modification enzymes will be the subject of the following section.

## Determinants of Specificity in tRNA Modification Enzymes

**Figure 2:**
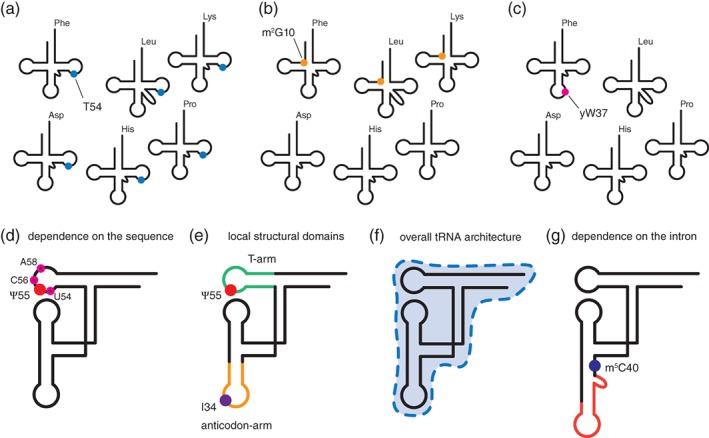
Determinants of specificity in tRNA modification enzymes. (a–c) Modification enzymes can be classified into three classes depending on their degree of specificity with respect to tRNA identities. Examples are drawn based on the specificity found in the yeast *S. cerevisiae*. (a) The first class consists of modification enzymes that modify all tRNAs with the correct target nucleotide, such as Trm2 introducing T54 in all cytoplasmic tRNAs in yeast. (b) The second class consists of modification enzymes that modify only a subset of tRNAs having the correct target nucleotide, such as Trm11/Trm112 introducing m^2^G10 in approximately half of the tRNAs in yeast. (c) The third class consists of modification enzymes that modify only a single tRNA species. For instance in yeast, the complex modification wybutosine (yW) is found uniquely at position 37 of tRNA^Phe^. (d–g) General principles controlling substrate recognition by tRNA modification enzymes. These general principles include (d) dependence on the RNA sequence, (e) dependence on local structural domains, (f) dependence on the overall tRNA architecture, and (g) dependence on the presence of an intronic sequence. A combination of these different principles determines the actual specificity of tRNA modification enzymes, which often involves both a certain degree of sequence specificity and structural determinants.

The features in the tRNA substrate that control whether a given position in its sequence is modified or not are collectively referred to as “determinants of specificity.” From a general perspective, modification enzymes can be classified into three classes depending on their degree of specificity with respect to tRNA identities (Fig. 2a–c). The first class includes modification enzymes with an activity dictated by the chemical nature of the target nucleotide. For instance in *S. cerevisiae*, all the cytosolic tRNAs having a U at position 54 are modified at this position to m^5^U54 also designated T54 (Fig. 2a). This almost completely conserved modification incorporated by Trm2 in yeast, TrmA in *E. coli* and TrmFO in *B. subtilis* and *T. thermophilus* 30, 31, 32, 33, is at the origin of the name given to this subdomain of tRNAs, namely the T‐arm (Fig. 1b). This class also includes the Tad2/Tad3 heterodimer, which introduces the I34 modification in all the yeast cytosolic tRNAs having an A at position 34 34, 35. In addition, the Trm5 and TrmD enzymes that modify all tRNAs having a G at position 37 to m^1^G37, in yeast and *E. coli*, respectively, belong to this class 36, 37. m^1^G37 is further modified into yW in yeast tRNA^Phe^ (see below).

The second class consists of enzymes that modify only a subset of tRNAs having the correct target nucleotide at the position to be modified. This class includes the Trm11 enzyme from yeast, which is responsible for the synthesis of m^2^G10 in tRNAs in association with the ubiquitous partner protein Trm112 that is also required for the function of other methyltransferases 38, 39, 40, 41. The m^2^G10 modification is found in approximately half of the tRNAs, although all yeast tRNAs have a G at position 10 (Fig. 2b). Other yeast enzymes belonging to this class are the Trm10 enzyme, which introduces the m^1^G9 modification in approximately half of the cytosolic tRNAs having a G at position 9 42, 43, the Trm6/Trm61 heterodimer, which introduces the m^1^A58 modification in about two‐thirds of cytosolic tRNAs (all having an A at this position) 18, 34, 44, and the Trm3 enzyme, which introduces the Gm18 modification in about one‐third of cytosolic tRNAs (all having a G at this position) 45. The m^1^A58 modification is not found in *E. coli*, but in prokaryotes such as *Thermus thermophilus* or *Pyrococcus abyssi*, the m^1^A58 modification is introduced by tetrameric TrmI enzymes 46, 47, 48 with a certain degree of sequence specificity 49. This implies that probably only a subset of tRNAs are substrates of these enzymes like for Trm6/Trm61, although this point has not been yet systematically analyzed, the complete patterns of tRNA modifications in these species being sparse or inexistent 8. In *E. coli* and other bacteria such as *Aquifex aeolicus*, the Gm18 modification is introduced by the TrmH enzyme that modifies only a subset of tRNAs, like the Trm3 enzyme, whereas in other bacteria such as *T. thermophilus*, all tRNAs are substrate of the TrmH enzyme 50. In *E. coli*, this class of modification enzymes also includes TrmJ, which catalyzes the Um32 and Cm32 modifications in the anticodon 51, 52. Interestingly, the specificity of an archaeal homolog of TrmJ in *Sulfolobus acidocaldarius* is different from the one of *E. coli* and methylates the ribose of cytidines and not uridines 53. An important point that is manifest in these examples is that the understanding of the determinants of specificity for this class of modification enzymes is very difficult to appreciate and generalize as the behavior of a given enzyme in one organism might not apply to other species, even though their enzymes might look very similar.

The third class includes modification enzymes that are highly specific to the tRNA identity because they modify a single tRNA species. An example in yeast is the enzyme that introduces the complex modification yW uniquely at position 37 of tRNA^Phe^ and not in the other tRNAs having a G at this position (Fig. 2c). This type of highly specific modification enzymes also include the enzyme catalyzing the 2′‐O‐ribosyl phosphate addition to A64 in yeast initiator tRNA_i_^Met^ 54, the tRNA^His^ guanylyltransferase Thg1 that adds the G_−1_ residue unique to tRNA^His^ 55, 56, Dnmt2 that introduces the m^5^C38 modification specifically in tRNA^Asp^ or tRNA^Glu^ depending on the organism 57, 58, and the acetyltransferase TmcA that introduces the ac^4^C34 modification in *E. coli* Elongator tRNA^Met^ 59. The enzymes of this class are often highly sequence‐specific, such as for instance Tgh1 that adds the G_−1_ residue to tRNAs possessing a histidine GUG anticodon 55.

All the tRNA modification enzymes recognize and distinguish their tRNA substrates based on the RNA sequence (Fig. 2d), the overall tRNA architecture (Fig. 2e), local structural domains (Fig. 2f), and the presence or the absence of an intronic sequence (Fig. 2g), which altogether form the determinants of specificity (Table 1).

**Table 1:** Determinants of specificity in tRNA modification enzymes

Enzyme^a^	Modification	Sequence	Local structural domains	Overall tRNA architecture	Intron	References
TruB (Ec)	Ψ55	**●**	**●**			60, 61
TrmB (Aa)	m^7^G46	**●**	**●**			62, 63
Trm8/Trm82 (Sc)	m^7^G46		**●**			64, 65
TadA (Ec, Sa)	I34		**●**			66, 67
TrmL (Ec)	Cm34/Um34		**●**			68
TrmO (Ec)	m^6^t^6^A37	**●**	**●**			69
TrmJ (Ec)	Cm32/Um32			**●**		52
Trm11 (Tk)	m^2^G10/m^2^_2_G10			**●**		70
Trm11/Trm112 (Sc)	m^2^G10			**●**		41
Trm1 (Sc, Pf, Aa)	m^2^_2_G26			**●**		71, 72, 73, 74
Trm4 (Sc)	m^5^C34, m^5^C40				**●**	75, 76
Pus1, Pus7 (Sc)	Ψ34, Ψ35, Ψ36				**●**	75, 76

a Organisms are given in parenthesis. Ec: *Escherichia coli*; Aa: Aquifex aeolicus; Sc: *Saccharomyces cerevisiae*; Sa: *Staphylococcus aureus*; Pf: Pyrococcus furiosus; Tk: Thermococcus kodakarensis.

### Sequence Recognition

The pseudouridylation catalyzed by the bacterial TruB enzyme to yield Ψ55 in the T‐arm of tRNAs is primarily controlled by sequence‐specific features in the T‐loop, with U54, C56 and especially A58 being essential for recognition and catalysis (Fig. 2d) 60, 61. In addition, the stem‐loop structure formed by the T‐arm is necessary and sufficient for Ψ55 modification by TruB, which therefore depends on a local structural domain and not on the overall tRNA structure (Fig. 2e). Similarly, tRNA recognition by the bacterial TrmB enzyme from *A. aeolicus* that introduces m^7^G46 in most tRNAs with a short variable region (Fig. 1b), is achieved via a combination of sequence and local structure determinants 62. The Trm8/Trm82 heterodimer that catalyzes the same reaction in yeast has stricter recognition requirements than TrmB and needs both the T‐ and D‐arms but not a fully intact tRNA for efficient catalysis 64, 65.

### Local Structural Domains Versus Overall tRNA Architecture Recognition

Altering key tertiary interactions essential for the proper three‐dimensional folding of tRNAs showed that some modification enzymes require an intact tRNA architecture, whereas others remain active on imperfectly folded tRNAs 77. A comprehensive study of yeast tRNA^Asp^ microinjected in *Xenopus laevis* oocytes showed that most modifications enzymes targeting the branch of the L formed by the T‐arm and accepting stem (Fig. 1c) do not need an intact tRNA as substrate, whereas most of the modification enzymes targeting the other branch formed by the D‐arm and anticodon‐arm (Fig. 1c) are sensitive to changes in the global tRNA architecture 77. However, this classification only provides a general trend regarding the specificity of modification enzymes families.

Many studies have characterized modification enzymes for which local structural domains are the main determinants of specificity (Fig. 2e). For instance, tRNA recognition by the bacterial enzyme TadA that converts adenosine into inosine at position 34 of the anticodon loop does not depend on the global tRNA architecture but on the local anticodon stem‐loop structure (Fig. 2e) 66, 67. Similarly, *E. coli* TrmL and TrmO are active on anticodon stem‐loops 68, 69.

Conversely, several modification enzymes for which the substrate recognition requires an intact tRNA architecture were identified (Fig. 2f). The tRNA recognition mode of some of these enzymes has been characterized structurally, which shed light on the molecular basis that makes the intact tRNA architecture required for binding and activity. For instance, *E. coli* TrmJ requires an intact elbow and anticodon‐loop regions for methylation, whereas the size of the acceptor stem can be reduced without affecting its catalytic activity 52. Conversely, the archaeal Trm11 requires an intact elbow and accepting region in the tRNA substrate. A docking model of the tRNA/Trm11 complex suggested that substrate recognition and catalysis is achieved by a molecular ruler mechanism in which the distances between the active site and both the 3′‐CCA end and the variable region are precisely defined 70. A similar tRNA/enzyme model was proposed for the orthologous yeast Trm11 enzyme that acts as a heterodimer with the Trm112 protein. However, in this case, additional contacts are formed between the tRNA anticodon‐loop and the Trm112 partner 41. Another family of enzymes sensitive to the overall tRNA architecture is constituted by Trm1 enzymes, which catalyze m^2^_2_G26 formation in the tRNA core. Although all enzymes from this family are sensitive to alterations in the tRNA three‐dimensional structure, the eukaryotic and archaeal enzymes recognize primarily the D‐arm and the variable region, whereas the bacterial enzyme from *A. aeolicus* has recognition sites in the tRNA elbow and T‐arm regions 71, 72, 73, 74.

### Intron Recognition

A particular set of modifications enzymes sensitive to local structural domains consist of enzymes requiring the presence of the intron in the tRNA substrate (Fig. 2g). As expected, the corresponding modifications are located in the anticodon‐loop region 75. In yeast, the modifications enzymes that require the presence of the intron catalyze the formation of m^5^C34, Ψ34, Ψ35, Ψ36, and m^5^C40 75, 76, whereas those that require the prior removal of the intron include the enzymes introducing Gm18, Cm32, Ψ32, m^1^G37, yW37, i^6^A37, and Um44 in tRNAs 75, 76, 78.

### Multispecific Modification Enzymes

Multispecific modification enzymes add a new flavor to the question of the specificity in tRNA modifications. Multispecific enzymes can be divided into two groups, modification enzymes that modify several positions in tRNA substrates, or modification enzymes that modify tRNA substrates as well as non‐tRNA substrates. Multispecific enzymes that modify several positions in tRNAs include archaeal TrmI that introduces m^1^A on both A57 and A58 47, bacterial Trm1 that introduces m^2^_2_G on both G26 and G27 74, Trm4 that introduces m^5^C at multiple positions 79, 80, pseudouridine synthases Pus1, Pus3, and TruA that introduce Ψ at multiple positions 81, 82, 83, dihydrouridine synthases Dus1 and Dus4 in *S. cerevisiae* and DusA in *E. coli* that introduce D at positions 16 and 17 for Dus1 and 20a and 20b for Dus4 and DusA 84, 85, and the archaeal ArcTGT from *Thermoplasma acidophilum* that catalyzes the formation of preQ_0_ at both positions 15 and 13 in the D‐arm of tRNAs 86. In the case of the multispecific enzyme Trm7 from yeast that introduces Cm32 and Nm34 in the anticodon loop, auxiliary subunits Trm732 and Trm734 regulate the site of modification by associating with the catalytic subunit 34, 87. Interestingly, some enzymes from the Trm10 family can methylate both adenosine and guanosine at position 9 of tRNAs 88, 89, 90. Multispecific enzymes that modify tRNAs as well as other RNA substrates include bacterial TrmA from *E. coli* that has been shown to also introduce m^5^U54 in tmRNA 91, bacterial RlmN from *E. coli* that catalyzes m^2^A modification both at position 2,503 of 23S rRNA and at position 37 of several tRNAs 92, and eukaryotic TRMT61B that catalyzes m^1^A formation both at position 58 of mitochondrial tRNAs and at position 947 of human mitochondrial 16S rRNA 93. Finally, it is worth mentioning that some RNA modifications found in mRNAs are likely introduced by tRNA modification enzymes 94, 95. This is the case for Ψ and m^5^C modifications, which are introduced by members of the Pus and Trm4 families, that already have promiscuous activities within tRNAs (see above). Overall, the fact that many tRNA modification enzymes do not require an intact tRNA architecture for activity but are instead dependent on specific sequence and local structures (Fig. 2d,f and text above) suggests that the same modifications may be introduced in mRNAs by tRNA modifying enzymes.

## Prior Modifications in tRNA Can Influence the Introduction of New Modifications

**Figure 3:**
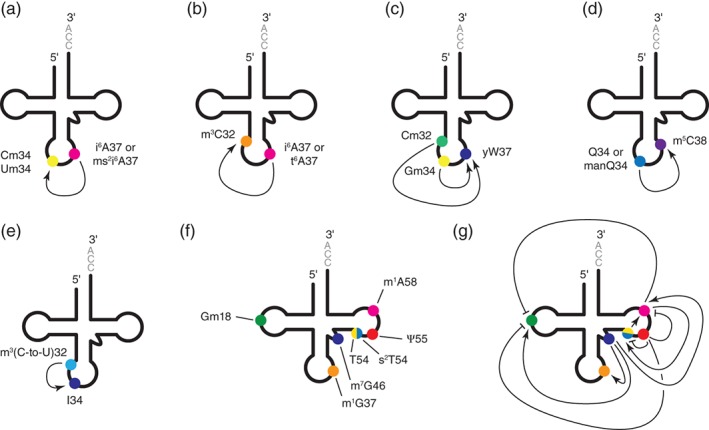
Modification circuits in tRNAs. (a–e) Modification circuits found in the anticodon‐loop region. (a) i^6^A37 or ms^2^i^6^A37 stimulate Um34 and Cm34 formation in *E. coli* tRNA^Leu(UAA)^ and tRNA^Leu(CAA)^. (b) i^6^A37 stimulates m^3^C32 formation in tRNA^Ser^ of *S. pombe* and *S. cerevisiae* and t^6^A37 stimulates m^3^C32 formation in tRNA^Thr^ of *S. cerevisiae*. (c) Cm32 and Gm34 stimulate wybutosine yW37 formation in eukaryotic tRNA^Phe^. (d) Queuosine Q34 or mannosyl–queuosine manQ34 stimulate m^5^C38 formation in tRNA^Asp^ of several organisms (see text). (e) m^3^U32 originating from m^3^C32 stimulates I34 formation in *T. brucei* tRNA^Thr(AGU)^. (f, g) An intricate modification network in *T. thermophilus*. (f) The modifications involved in the modification network in *T. thermophilus* are represented on the cloverleaf representation of tRNA with filled colored circles. Gm18 is shown in green, m^1^G37 in orange, m^7^G46 in purple, T54 and s^2^T54 as half‐filled circles in yellow and blue, respectively, Ψ55 in red, and m^1^A58 in pink. (g) An intricate modification network in *T. thermophilus* enables the adaptation of tRNA modifications in response to temperature changes. The same color code is used as in panel (f). Arrows indicate stimulatory effects and blunted lines inhibitory effects.

In addition to the determinants of specificity described in the previous section (Fig. 2), the introduction of several modifications in tRNAs are influenced by the presence of existing modified nucleotides. In the literature, this aspect of RNA modification is called “modification circuits” to emphasize the fact that it connects modifications with one another and obviously drives a defined order in the introduction of these modifications 96. The term “network” is also sometimes used to underline the fact that modifications may be interconnected with several others 97. Most of the well‐documented examples of modification circuits occur in the anticodon‐loop region and involve modifications at position 34 or 37 or both, positions that are two of the most frequently modified in tRNAs, and at which most complex modifications are found (Fig. 3a–e) 8, 14. Although the exact reasons for which modification circuits exist in tRNAs and are mostly found in the anticodon‐loop region are not known, it was recently proposed that modifications introduced first act as additional recognition elements for other modifications, which provides the mean for adding modifications with considerable variation in the anticodon‐loop region, despite the lack of variability in its local sequence and structure 96. These modification circuits in the anticodon‐loop region include: the Um34 and Cm34 modifications introduced in *E. coli* on tRNA^Leu(UAA)^ and tRNA^Leu(CAA)^ by TrmL, which require prior formation of i^6^A37 or ms^2^i^6^A37 (Fig. 3a) 68, 98; the m^3^C32 modification introduced in yeast on tRNA^Ser^ and tRNA^Thr^ by Trm140, which is greatly stimulated by the prior formation of i^6^A37 or t^6^A37 (Fig. 3b) 99, 100; the complex wybutosine yW37 modification in the anticodon loop of eukaryotic tRNA^Phe^, which requires prior Cm32 and Gm34 modifications (Fig. 3c) 87, 101; the m^5^C38 modification introduced in mammals, *Drosophila melanogaster*, *Arabidopsis thaliana*, and *S. pombe* on tRNA^Asp^ by the Dnmt2 family of enzymes, which is greatly stimulated by prior queuosine Q34 or mannosyl–queuosine manQ34 modifications (Fig. 3d) 57, 102, 103; and the I34 modification introduced in *Trypanosoma brucei* on tRNA^Thr(AGU)^ by ADAT2/ADAT3 heterodimer, which is stimulated by prior deamination of C32 leading to U32, which is itself stimulated by the prior methylation of C32 leading to m^3^C32 (Fig. 3e) 104, 105. In all these cases, the precise molecular mechanisms that define these modification circuits are not yet fully understood, but it is reasonable to conceive that the prior modification might either act as a direct recognition determinant for the subsequent modification enzyme or alter the local structure of the anticodon‐loop region, thereby helping to present the appropriate structure to the subsequent modification enzyme. Modified bases in the anticodon‐loop indeed reinforce its U‐turn anticodon‐loop structure. Modifications at positions 32 and especially 37 prevent formations of base pairs between nucleosides 32–38 and 33–37, thereby enabling canonical anticodon‐loop folding 12, 106, 107.

In addition to these modification circuits in the anticodon‐loop region, several circuits involving modifications in the tRNA core have been described in *T. thermophilus* (Fig. 3f,g) 108. *T. thermophilus* is an extreme thermophilic bacteria that can grow at temperature ranging from ~50 to 80 °C, and in which changes in external temperature conditions result in changes in the modification level of several modifications in the tRNA core. These variations in the modification content of tRNAs depending on the external temperature have been shown to be linked to an adaptation of protein synthesis to temperature change 109. Interestingly, this adaptation is not controlled by transcriptional and/or translational regulations, but is established via modification circuits. These modification circuits in the tRNA core include: the m^1^A58 modification, which is stimulated by T54 110; the s^2^T54 modification from T54, which is stimulated by m^1^A58 111; the Gm18, m^1^G37, and m^1^A58 modifications, which are stimulated by m^7^G46 112; the Gm18, s^2^T54, and m^1^A58 modifications, which are negatively regulated by Ψ55 113; and the Gm18 modification, which is negatively regulated by m^1^A58 110 (Fig. 3g). These modification circuits regulate the extent of modifications in response to temperature change. For instance, at elevated temperature, the presence of m^7^G46 favors the introduction of Gm18 and m^1^A58, which further stimulates formation of s^2^T54, the two latter modifications being essential for survival at high temperature (~75–80 °C) 112. In contrast, at lower temperature, the Ψ55 modification inhibits formation of Gm18, m^1^A58, and s^2^T54, which maintains a sufficient flexibility of tRNAs at low temperatures, an essential feature for *T. thermophilus* survival at low temperature (~50–55 °C) 113. Such a mechanism of regulation, which does not include any transcriptional or translational regulatory steps and is not limited to *T. thermophilus*, makes the response of the network to environmental changes very rapid. Here, modification circuits are controlling variations in the modification content of tRNAs in response to temperature changes, but alteration of the modification status of tRNAs in response to environmental changes is not limited to *T. thermophilus* and may not necessarily involve circuits of modifications. The dynamic changes in tRNA modifications will be described in the next section.

## Variations in tRNA Modifications in Response to Environmental Changes

**Figure 4:**
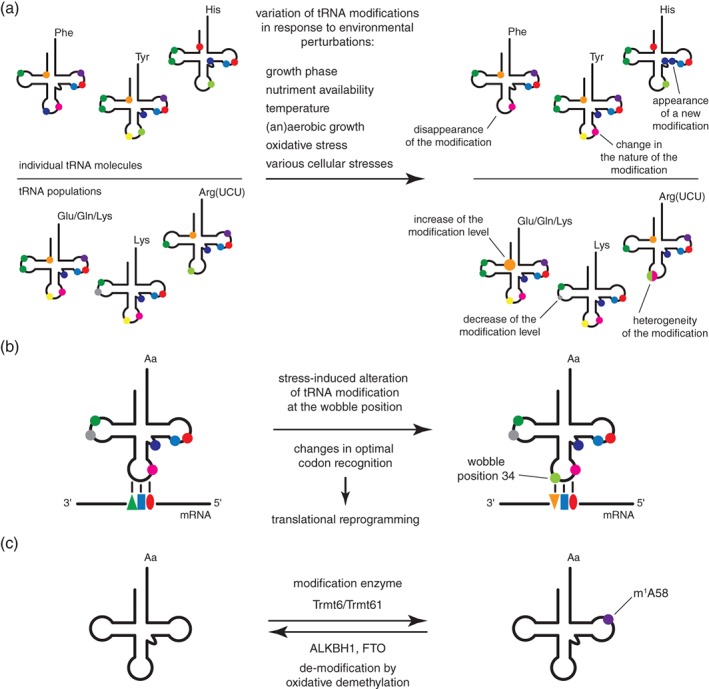
Dynamic changes in tRNA modification levels. (a) Variations of tRNA modifications in response to environmental perturbations. In response to various environmental or cellular factors, such as growth phase, nutriment availability, temperature, aerobic versus anaerobic growth, oxidative stress, or various cellular stresses, the levels and/or the nature of certain modifications can change in tRNAs. Variations occur at the level of individual tRNA molecules (disappearance of a modification, appearance of a new modification, and change in the nature of the modification). These variations collectively impact the level of modifications in tRNA populations, such as particular isoacceptors (e.g., tRNA^Lys^) or isodecoders (e.g., tRNA^Arg(UCU)^). In a certain tRNA population, these variations may appear in the form of increased or decreased modification levels, or may introduce modification heterogeneities at certain positions. (b) Stress‐induced alteration of tRNA modification at the wobble position can lead to translational reprogramming. Stress‐induced alteration of tRNA modification at the wobble position 34 changes the optimal codon recognition of the given tRNA and thereby upregulates the formation of critical stress‐response proteins encoded by mRNA transcripts enriched in this particular codon adapted to the altered wobble modification. (c) Variations in tRNA modifications and active demodification processes. The m^1^A58 modification, introduced in human by the Trmt6/Trmt61 modification enzyme, can be actively removed by the demodification enzymes ALKBH1 and FTO via an oxidative demethylation process.

Although it has been commonly assumed that tRNA modifications are constitutively introduced as static decoration on every tRNA of the cell, there is now established and continually growing evidence indicating that the level and/or the nature of certain modifications in a tRNA population can change dynamically in response to several environmental factors, such as cellular stresses, temperature, or nutriment availability 11. At the level of individual tRNA molecules, these variations consist in the appearance, disappearance, or change of the nature of the modification (Fig. 4a). These variations collectively have an impact on the global level of modifications in tRNA populations. For instance, some subgroups such as particular isoacceptors or isodecoders might be affected differently (Fig. 4a).

### Factors Causing Variations in tRNA Modifications

Several factors, including growth phase, growth rate, growth medium, (an)aerobic growth, and temperature, were reported to affect the levels and/or the nature of tRNA modifications (Table 2). For instance, the level and the nature of modifications have been shown to vary between different growth phases in *B. subtilis* 114, 115. In addition, the synthesis of Q34 from its derivative oQ requires vitamin B_12_, which is only synthetized in *S. typhimurium* under anaerobic conditions, provided that cobalt ions, glycerol as the carbon source, and fumarate as the electron acceptor are present. Therefore, oQ34 is present in tRNAs from bacteria grown in glucose salt medium lacking vitamin B_12_, whereas Q34 is formed in bacteria grown in rich media 120. Furthermore, a meticulous study of the tRNA modification pathways of wobble U34 in the *E. coli* tRNAs that are substrates of MnmE/MnmG and MnmC modification enzymes, revealed that the growth conditions, namely growth medium and growth phase, affect the enzymatic pathway leading to the final modifications 126. Changes in tRNA modifications in response to external factors and growth conditions are not restricted to prokaryotes and the levels of many modifications are sensitive to changes in temperature in *S. cerevisiae*, with for instance the thiolation of U34 being impaired at elevated temperatures (>30 °C), a temperature at which tRNAs therefore display mcm^5^U34 instead of mcm^5^s^2^U34 122. In *S. cerevisiae*, additional m^5^C modifications were shown to be specifically introduced on tRNA^His^ at position 48 and 50 in response to several growth arrest conditions, yet the significance of these additional m^5^C remains unclear 127. Also in human, several types of cancer cell lines were shown to contain tRNAs with perturbed contents of modified bases 128. The tumor‐specific tRNA species are usually hypomodified, with for instance reduced wybutosine yW37 modification of tRNA^Phe^ 124 and reduced levels of queuosine Q34 of tRNA^Asp^ 125.

**Table 2:** Factors causing variations in tRNA modifications

System description			Growth phase			Growth medium		Growth conditions				
Organism^a^	tRNA	Modification	Exponential phase	Stationary phase	Growth rate	Rich medium	Glucose salt medium	Aerobic growth	Anaerobic growth	Temperature	Tumors	References
Bs			**+** b	**++**								114
Bs	tRNA^Tyr^		i^6^A37	ms^2^i^6^A37								115
Ec		ms^2^i^6^A37	**●**									116
Ec		Q34	**●**									117
Ec	tRNA^Leu^_1_, tRNA^Gly^_2&3_, tRNA^Pro^_1_	s^4^U8			**●**							118
Bs	tRNA^Phe^					Gm34	G34					119
Bs	tRNA^Phe^					ms^2^i^6^A37	i^6^A37					119
St						Q34	oQ34					120
St								ms^2^io^6^A37	ms^2^i^6^A37			121
Tt, Mb, Sh, Sc										**●**		12, 112, 113, 122, 123
Hs	tRNA^Phe^	yW37									**–** c	124
Hs	tRNA^Asp^	Q34									**–** c	125

a Bs: Bacillus subtilis; Ec: *Escherichia coli*; St: Salmonella typhimurium; Tt: Thermus thermophilus; Mb: Methanococcoides burtonii; Sh: Stetteria hydrogenophila; Sc: *Saccharomyces cerevisiae*; Hs: Human.

b Hypomodified in the exponential phase compared to the stationary phase.

c Hypomodified in tumors.

### Alteration of Modifications at the Wobble Position and Translational Reprogramming

Although the roles of these various alterations in the levels of modifications are in most cases unknown, a recent concept has emerged that provides a convincing explanation regarding the role of modification alterations in response to environmental changes for a subset of modifications located at the wobble position 129. In this model, stress‐induced alteration of tRNA modification at the wobble position promotes a translation regulation to increase the formation of critical stress‐response proteins encoded by mRNA transcripts with a particular codon bias adapted to the altered wobble modification (Fig. 4b). A quantitative analysis of modifications in tRNA by HPLC‐coupled mass spectrometry showed that dynamic changes of these modifications in response to various cellular stress conditions are widespread and stress‐specific 130. For instance, Cm, m^5^C, and m^2^_2_G modifications increase in response to H_2_O_2_ exposure but decrease or are unaffected by exposure to the methylating agent MMS. In subsequent studies, some specific changes of tRNA modification levels were shown to drive selective translation of codon‐biased mRNAs. For instance, in *S. cerevisiae* exposed to H_2_O_2_, an increase in the proportion of tRNA^Leu(CAA)^ containing m^5^C at the wobble position promotes selective translation of mRNA from genes enriched in the TTG codon, including expression of Rpl22a, a ribosomal protein that contributes to the oxidative stress survival response 131. Similarly, in *S. pombe*, the mcm^5^s^2^U modification introduced for its cm^5^ part by Elongator at the wobble position of tRNA^Lys(UUU)^ was shown to be critical for efficient translation of certain stress‐induced mRNAs from genes enriched in the AAA codon because strains lacking functional Elongator displayed higher stress sensitivity to H_2_O_2_ 132, 133. Later reports highlighted other facets of the role of tRNA wobble mcm^5^s^2^U34 modifications in translation of codon‐biased mRNAs, where either the methylation catalyzed by Trm9/Trm112 heterodimer leading to mcm^5^U from cm^5^U, or the thiolation introduced by the URM1 pathway leading to (mcm^5^)s^2^U34 were shown to promote effective translation of mRNAs enriched in the AGA and GAA codons for the methyl group and in the AAA and GAA codons for the thiol group 134, 135. Furthermore, dynamic changes in the level of U34 thiolation in response to elevated temperature in *S. cerevisiae* were proposed to contribute to the heat shock response by reducing translation of specific genes 135. In addition, an increase of wobble cmo^5^U34 in tRNA^Thr(UGU)^ of *Mycobacterium bovis* in response to hypoxia was reported to promote translation of specific transcripts enriched in the ACG codon, including the DosR master regulator of hypoxia, here again linking stress‐induced tRNA modification changes to translation reprogramming 136. Finally, it is worth to briefly mention that tRNA modifications have been linked to stress response via a seemingly unrelated mechanism implicating tRNA stress‐induced cleavage and the formation of tRNA‐derived small‐RNAs, with some tRNAs becoming more sensitive to endonucleolytic cleavage in the absence of m^5^C modifications 4, 137.

## Variations in tRNA Modifications and Active demodification Processes

In all RNA families, variations in the levels of modifications in a given tRNA in response to environmental changes or other factors can be achieved via (i) changes in the expression or the activity of modification enzymes; (ii) alteration of RNA synthesis or degradation; (iii) involvement of demodification enzymes. Considering the different stability and turnover rates of each RNA family (for instance tRNAs and mRNAs), the mechanisms leading to variations in modification levels might be different. At present, the origin of the variations in the modification content in RNAs and especially mRNAs, which have been collectively described under the term “epitranscriptomics” is under intense investigations 138. A fascinating mechanism for achieving these dynamic changes of RNA modifications consists in the active removal of specific modifications by demodification enzymes. A few examples of active demodification processes that have been reported in tRNAs will be described below.

Recently, human ALKBH1 and the fat mass and obesity‐associated protein (FTO) were reported to catalyze demethylation of m^1^A in tRNAs (Fig. 4c) 139, 140. Both proteins belong to the AlkB family of proteins that consist of dioxygenases that use a non‐heme Fe(II) and α‐ketoglutarate to catalyze biological oxidation. The prototypical member of this family is the *E. coli* AlkB protein that functions in DNA/RNA alkylation damage repair by removing alkyl groups on nucleic acids 141, 142, 143. Interestingly, ALKBH1 displays a tRNA‐binding domain related to the ones found in eukaryotic tRNA ligases and binds to m^1^A58‐containing tRNAs inside cells 139. Concerning tRNAs, ALKBH1 and FTO display a pronounced preference for the demethylation of m^1^A over other common methylated nucleotides such as m^7^G, m^5^C, and m^3^C for ALKBH1, and m^7^G, m^5^C, m^2^G, and m^1^G for FTO. However, ALKBH1 and FTO display a different nucleotide‐specificity toward other nucleic acid substrates. In DNA, ALKBH1 and FTO catalyze demethylation of m^3^C and m^3^T, respectively 144, 145. FTO was also reported to catalyze demethylation of m^3^U and m^6^A in mRNA 146, 147, and to display a slight tRNA m^3^C demethylation activity in vitro 140. As FTO preferentially catalyzes m^1^A demethylation in stem‐loop structures mimicking the T‐arm of tRNAs, m^1^A58 is most likely the primary demethylation target of FTO in tRNAs (Fig. 4c) 140. ALKBH1 and FTO expression is increased in response to glucose deprivation, which decreases the m^1^A content of certain tRNAs and most probably the stability of tRNA_i_^Met^, thereby inhibiting translation initiation. This mechanism might contribute to globally repress translation on glucose deprivation 139, 140.

These examples highlight the importance of tRNA modification levels in the global control of fundamental cellular functions such as translation and reveal that a demodification enzymatic activity alters modification levels in tRNAs. It remains to be discovered whether demodification processes are limited to m^1^A modifications or whether theses aspects of reversible modifications apply to other types of modifications in tRNAs.

## CONCLUDING REMARKS

The picture that emerges from this review is that the introduction of modifications in tRNAs is amazingly complex. Not so long ago, it was thought that modifications are introduced in tRNAs showing the correct sequence and structural requirements as static chemical decorations, and their importance for optimal tRNA function was clearly well appreciated. At the same time, the concept that regulation of tRNA biological function could be mediated by its modifications was accepted, but the exact mechanisms by which these regulations could be achieved were missing. Recently, answers to these questions were provided by reports showing that (i) modification circuits establish functional connections between different modifications in tRNAs; (ii) modification levels can change dynamically in response to environmental perturbations such as cellular stresses, which may lead to translational reprogramming and translation of specific stress‐response genes; (iii) activation of demodification enzymes can remove modifications thereby altering tRNA function in response to defined stimuli. All these aspects have contributed to turn the spotlight on tRNA modifications in the recent years. Although the different aspects influencing nucleotide modifications in tRNAs have been classified and presented in different parts of this review, it is worth mentioning that all these aspects are deeply interconnected. For instance, modification circuits are just another layer adding to the determinants of specificity of the enzymes. In addition, modification circuits can alter the levels of tRNA modifications in response to environmental changes, with primary variations directly responding to defined stimuli while other ones being a consequence of the interplay between modifications. Finally, dynamic changes in the levels of tRNA modifications, in response to changes in the cellular conditions, can be mediated by active demodification processes. All things considered, the tRNA modification field has clearly entered an exciting period, and impressive new regulatory functions of modifications surely await to be discovered.
